# Effects of Parenting Styles on Self-Regulated Learning and Academic Stress in Spanish Adolescents

**DOI:** 10.3390/ijerph16152778

**Published:** 2019-08-03

**Authors:** María C. Fuentes, Rafael García-Ros, Francisco Pérez-González, Dolores Sancerni

**Affiliations:** 1 Department of Methodology of the Behavioral Sciences, Faculty of Psychology, University of Valencia, 46010 Valencia, Spain; 2 Department of Developmental and Educational Psychology, Faculty of Psychology, University of Valencia, 46010 Valencia, Spain

**Keywords:** parenting styles, school adjustment, academic stress, self-regulated learning, Spanish adolescents

## Abstract

Research has repeatedly highlighted the important influence of parental socialization styles on children’s psychosocial adjustment. However, previous studies about their effects on school adjustment have traditionally addressed a limited set of indicators, such as academic achievement or self-concept, which should be broadened in order to increase our level of knowledge about this topic. Thus, the aim of the present study was to analyze the relationships between parenting styles and other relevant school adjustment criteria (self-regulated learning and academic stress) in adolescence. The study participants were 437 Spanish adolescents (44.7% men) from 12 to 18 years old (*M* = 14.55, *SD* = 1.80) who were enrolled in high school. A multivariate factorial design (parenting × sex × educational level) was used for each set of criteria. The results are consistent with previous research, showing that the indulgent style was related to better school adjustment during adolescence, evaluated through self-regulated learning and academic stress, thus increasing the available evidence about the influence of parenting styles in this setting. Additionally, this relationship remains invariant with regard to sex and the educational level of the participants in the study. Therefore, this study highlights the importance of parenting practices related to high acceptance/involvement for the adequate school adjustment of Spanish adolescents.

## 1. Introduction

In recent years, empirical evidence has consistently shown the importance of family socialization in children’s adjustment, pointing out its influence on their psychosocial adaptation and wellbeing [1,2], with this influence lasting until adulthood [3,4].

The different patterns of practices that define parents in the socialization process have traditionally been studied using a theoretical model with two large theoretically independent dimensions, usually called acceptance/involvement and strictness/imposition in the literature [5,6]. Each dimension is represented by a set of parental practices that define these ways of behaving. Although the degree to which these practices are used can vary, the results of different studies have concluded that their relationships with the dimension they represent remains invariant [7]. Thus, on the one hand, the acceptance/involvement dimension is defined by the degree to which the parents make use of practices related to showing affection, support, and emotional involvement, as well as communication and the use of reasoning to modify poorly adjusted behaviors. On the other hand, the degree to which parents make use of practices related to parental firmness and imposition to establish limits defines the strictness/imposition dimension. From the confluence of these two orthogonal dimensions, four parenting styles emerge, each of which represents a particular form of approaching children’s socialization: the authoritative style (high acceptance/involvement and high strictness/imposition), the indulgent style (high acceptance/involvement and low strictness/imposition), the authoritarian style (low acceptance/involvement and high strictness/imposition), and, finally, the neglectful style (low acceptance/involvement and low strictness/imposition) [6,8,9].

The research in this area of study essentially attempts to determine which parenting style is related to better results in children’s adjustment. Although the empirical evidence is consistent in showing the lack of significant variations in the best parenting style depending on demographic variables such as the sex and age of the parents and the children [10,11,12,13], there have been important discussions about the optimal parenting style, depending on the cultural context in which this relationship is studied [14,15,16]. In this sense, as Darling and Steinberg noted (1993, p. 487), “particularly pressing issues are the variability in the effects of parenting style as a function of the child’s cultural background”. More current evidence highlights the key role of cultural context in the relationship between parenting and developmental outcomes [5,15,17]. Along general lines, since the first studies carried out in Anglo-Saxon contexts, the style characterized by high warmth and high strictness, the authoritative style, has consistently been associated with better psychosocial adjustment [8,9,18,19]. More recent studies carried out in this cultural context continue to find the authoritative style to be the best parenting strategy. Thus, children from authoritative families obtain higher scores on adjustment indicators such as self-esteem, satisfaction with life, happiness [20,21], academic achievement [15,22], resilience [23], and the use of adaptive strategies [24,25], and lower scores on indicators of poor adjustment, such as anxiety and depression [2,20], behavior problems, substance use [26,27,28], and weight-related problems [29,30].

However, the results of different studies carried out in other cultural contexts suggest that this combination of high warmth and high strictness is not always related to the best results for children’s adjustment. On the one hand, some studies carried out in Anglo-Saxon contexts with minority ethnic groups (Afro-Americans [31,32]; Asian-Americans [33,34]) and in Arabic cultures [35,36] suggest that the parenting style characterized by high parental strictness and no affection, the authoritarian style, is an optimal socialization strategy, as these authoritarian practices are culturally associated with a responsible education and commitment to the child’s care [37,38]. Hence, children from authoritarian families in these cultural contexts obtained the best results on different adjustment criteria. For example, they showed a lower probability of being involved in episodes of alcohol abuse [32], better academic achievement [15,33], and adequate mental health [38]. The same results can be extended to different studies carried out in conflictive neighborhoods, where these authoritarian practices are shown to be a protective factor for children’s adequate psychosocial adjustment [39,40,41,42].

On the other hand, another set of studies carried out in different countries in Europe and Latin America (Brazil [43,44], Peru [45], Portugal [46], Italy [47], Turkey [48], Mexico [49]) suggest that the parenting style characterized mainly by high warmth and low strictness, the indulgent style, is related to the best adjustment results in children, regardless of sex and the developmental stage in which they find themselves [12,17,50]. Thus, the results of these studies concluded that children from indulgent families obtain better or at least equal scores to children from authoritative families on various psychosocial adjustment criteria such as personal competence and internalization of values [51], self-esteem [52], satisfaction with life [43], doing physical exercise [53], and substance use [54].

Research carried out in Spain is framed within this line of results, suggesting that the indulgent parenting style is optimal for children’s adequate adjustment, with this result being consistent across sex and age of both children and parents [17,50,55,56,57,58,59,60,61]. This research provides extensive evidence by analyzing the relationships with different indicators of children’s psychological and social wellbeing [55,62,63], in which, despite the significant differences in these adjustment indicators by sex or age [13,64,65], the optimal parenting style did not vary according to these demographic variables. Thus, children from indulgent families obtained better results, or at least equivalent results to children with authoritative parents, on self-concept [13,58], emotional instability [58,66], social competence [60], empathy [64,66], internalizing values [3,17], child-to-parent violence [67], substance use [56,68], aggressive behavior [11,69,70,71], and sexist attitudes [72], among others.

These results are similar to those obtained for children’s school adjustment, which is understood as all the indicators related to academic success [73]. In this regard, the indulgent style is related to the best results on different indicators, such as academic achievement, mainly evaluated through the students’ grade-point average (GPA) and the number of grades repeated [57,66,68], academic self-concept [57], academic engagement [74], and problems with disruptive behavior in the classroom [61,68]. Some recent studies even highlight the indulgent style as a protector factor against bullying and cyberbullying problems [59,71,74,75].

Although these studies approach school adjustment through classic indicators such as academic achievement [76], self-concept, and behavior problems in school [77], the literature highlights the importance of understanding school adjustment from a multidimensional perspective that would include different cognitive, attitudinal, and behavioral factors [77]. Thus, other relevant educational indicators have received little attention from the research on the influence of parental socialization in Spanish adolescents. In this direction, this study focused on analyzing the relationship between parenting styles and two constructs that have received special attention in educational research in recent decades: self-regulated learning (SRL) and academic stress as an indicator of subjective wellbeing in adolescence.

SRL can be defined in the following way: “it is an active, constructive process whereby learners set goals for their learning and then attempt to monitor, regulate, and control their cognition, motivation, and behavior, guided and constrained by their goals and the contextual features in the environment” [78]. Thus, SRL integrates cognitive, affective-motivational, and behavioral dimensions, all of which are positively related to learning levels and academic results in adolescence, apart from being a basic competence to develop in high school. This study considered SRL dimensions that show a close relationship with school adjustment and academic achievement, including metacognitive strategies and self-efficacy for learning [79], time management strategies [80], exam anxiety [81], and the academic procrastination associated with students deficits in their academic self-regulation strategies [82,83,84].

Classic studies highlighted the significant effects of parenting styles on self-regulated learning [85,86], and also show inconsistencies in the results regarding the optimal parenting style depending on the cultural context in question. On the one hand, studies carried out mainly in Anglo-Saxon countries have concluded that children of authoritative parents make greater use of self-regulated learning strategies [24,85,86,87]. On the other hand, Erden and Uredi (2008) [88], in their study with Turkish adolescents, pointed out that students from authoritative and indulgent families used cognitive and metacognitive strategies more than students from authoritarian and neglectful families. In Spain, previous studies have also analyzed the significant influence of parenting styles on SRL in adolescence [89,90]. Thus, Cerezo, Casanova, de la Torre, and Carpio (2011) [89] concluded that students with authoritative and indulgent parents made greater use of self-regulated learning strategies than students from authoritarian and neglectful families. Suárez and Suárez (2019) [90] also concluded that students from authoritative and indulgent families showed better results than students from families with neglectful and authoritarian styles on learning strategies, time management strategies, and effort, at the same time showing significantly lower levels of avoidance goals of academic tasks. Finally, in the same line, Tur-Porcar, Jiménez-Martínez, and Mestre-Escrivá (2019) concluded that both parenting styles (authoritative and indulgent) were related to better perceived academic self-efficacy than the authoritarian and neglectful parenting styles [91].

Academic stress is one of the most widely used psychological constructs to evaluate adolescents’ subjective wellbeing in educational contexts. It is a generalized phenomenon in the different stages of the educational system that negatively affects students’ personal, emotional, and physical wellbeing [92,93,94], their levels of learning and academic achievement [95,96], and is related to early school dropout [97]. Thus, it is especially relevant to consider academic stress in adolescence, given that the school setting is one of the most significant contexts during this developmental stage and one of the sources of stress most frequently mentioned by adolescents [98,99]. However, studies that analyze the relationship between parenting styles and academic stress in adolescence are quite scarce, making it necessary to increase the research in this area. Along these lines, Wolfradt, Hempel, and Miles (2003) [100] concluded that the authoritative and indulgent styles are related to the use of more effective strategies for coping with stress in German adolescents. In addition, de la Torre, Casanova, García, Villa, and Cerezo (2011) [101] concluded that the indulgent style is related to lower levels of academic stress in Spanish adolescents, with the authoritarian style being associated with higher levels of stress.

Therefore, considering the influence of parental socialization on children’s adjustment and the scant research analyzing its relationships with other relevant and representative criteria of children’s school adjustment, the objective of the present study was to analyze which parenting style was related to higher levels of self-regulated learning—evaluated through cognitive indicators (metacognitive strategies and perception of lack of control over time), affective-motivational indicators (self-efficacy toward learning and exam anxiety), and behavioral indicators (academic procrastination)—with adolescents’ personal wellbeing in educational contexts—evaluated from a multidimensional perspective of academic stress (academic overload, classroom interaction, family pressure, and future expectations). Considering the results of previous research carried out in Spain, children from indulgent families expected to show results that are better or equivalent to those of children from authoritative families on the different criteria evaluated. Moreover, it was expected that this relationship would not vary depending on the sex and educational level of the participants.

## 2. Materials and Methods

### 2.1. Participants and Procedure

To carry out the study with a statistical power of 95% (1 − β = 0.95), an a priori estimation of the minimum sample size required was made [102], establishing the statistical inference of the Type I error rate at the conventional limit (α = 0.05) and a small–medium effect size (*f* = 0.20) [103] between parenting styles and the school adjustment criteria on the children. The results indicated that the sample would have to contain a minimum of 436 participants.

Based on this result, the final sample was composed of 437 high school students enrolled in seven public schools in the Valencian Community (Spain), which were previously selected at random from a full listing of all public educational centers [104]. Participants were 195 males (44.7%) and 241 females (55.3%) between 12 and 18 years old (*M* = 14.55 years, *SD* = 1.80). Regarding the educational level, 317 (72.1%) were studying Compulsory Secondary Education (CSE) (7th to 10th grades, 12 to 16 years old) and 122 (27.9%) Post-Compulsory Secondary Education (PSE) (Baccalaureate or mid-level vocational training, 17 and 18 years old). Because CSE in Spain is structured in two cycles (CSE-1—7th and 8th grades and CSE-2—9th and 10th grades), three educational levels were considered in this study: CSE-1 (159 students), CSE-2 (156 students), and PSE (122 students) [92,105,106,107,108].

Before undertaking the research, this study was reviewed and approved by the Ethics Committee of the University of Valencia (No. H1523870265031). Taking into account the rules of the Declaration of Helsinki, after obtaining the informed consent of the participants’ schools and families, the students filled out the instruments collectively and voluntarily during school hours the week before the first semester exams. The instruments were administered by collaborating psychologists from the research team in a 50-min session.

### 2.2. Instruments

To measure the parenting styles, the *Warmth/Affection Scale* and *Parental Control Scale* were administered, both integrated in the *Parental Acceptance-Rejection/Control Questionnaire* [109,110]. The *Warmth/Affection Scale* is composed of 20 items that evaluate the acceptance/involvement dimension, whereby the adolescents rate the frequency with which their parents are affectionate, responsive, and involved with them (e.g., “we talk about our plans and they take my opinions into account”; “they are interested in what I think, and they like me to talk about it”). The *Parental Control Scale* is composed of 13 items that evaluate the strictness/imposition dimension, where the adolescents rate the frequency with which they perceive that their parents monitor them in an imposing, firm, and demanding way (e.g., “they want to control anything I do”; “they are always telling me how to behave”). Both scales have a response scale ranging from 1 (“never”) to 4 (“always”), so that high scores indicate a high degree of acceptance/involvement and strictness/imposition. The internal consistency obtained for each scale was, respectively, 0.91 and 0.79.

Self-regulated learning was evaluated with different subscales from the Spanish adaptations of the *Motivated Strategies for Learning Questionnaire* (MSLQ) [111,112] and the *Time Behavior Management Scale* (TMBS) [106,113]. More specifically, the following MSLQ subscales were used in this study: *Metacognitive Strategies,* which consists of 6 items that evaluate the processes of planning, monitoring, and metacognitive regulation in an academic context (e.g., “I try to change the way I study in order to fit the course requirements and the instructor’s teaching style.”); *Self-Efficacy for Learning,* which consists of 7 items that evaluate the student’s confidence in his/her ability to perform the academic tasks (e.g., “I’m confident I can understand the most complex material in this course”); and *Test Anxiety,* which evaluates the cognitive and emotional components of test anxiety with 5 items (e.g., “when I take tests, I think of the consequences of failing”). Items on the different MSLQ subscales are rated on a seven-point scale from 1 (*never*) to 7 (*always*). Cronbach’s alpha obtained for each subscale was 0.71, 0.89, and 0.70, respectively. From the TMBS, the following subscales were used: *Perception of control over time,* which is made up of 5 items that evaluate the degree to which the subject perceives that he/she effectively controls and manages his/her time. Given the inverse nature of the factor, high scores indicate the sensation of lack of control over time, feeling overwhelmed by trivial tasks and details, dedicating a lot of time to secondary tasks, or taking on too many tasks and responsibilities (e.g., “I have to spend a lot of time on unimportant tasks”). *Academic Procrastination* has 7 items that evaluate the student’s voluntary delay of action on academic tasks despite expecting to be worse off for that delay (e.g., “I start studying for exams at the last minute”). The TMBS subscales are rated on a five-point scale ranging from 1 (*never/almost never*) to 5 (*always/almost always*). The internal consistency for each subscale was 0.73 and 0.75, respectively.

Academic Stress was evaluated with the *Questionnaire of Academic Stress in Secondary Education* (QASSE) [92]. The QASSE contains 24 items that describe everyday academic situations that can potentially produce stress. Subjects must respond by indicating the level of stress each situation produces using a Likert-type response scale ranging from 1 (very low) to 5 (very high). The questionnaire evaluates four dimensions: *Academic overload,* which consists of 9 items that evaluate the level of stress produced by the perception of lack of time and the feeling of being overwhelmed by the amount of academic work, as well as taking exams (e.g., “taking exams”), *Future Perspectives,* which contains 4 items that rate the stress produced by the anticipation of situations and possible future academic problems, such as choosing classes and electives, or finishing their studies in the stipulated time (e.g., “finishing 10th grade or 12th grade or Vocational Education”); *Classroom Interactions,* which includes 7 items that evaluate the level of stress produced in the interpersonal interactions and conflicts with classmates and teachers in the school setting (e.g., “problems or conflicts with classmates”); *Family Pressure,* which contains 4 items that rate the level of stress in the family environment produced by situations from the academic setting (e.g., “family discussions and conflicts caused by my studies”). The Cronbach’s alpha obtained in this study for each of the dimensions was 0.84, 0.73, 0.74, and 0.72, respectively.

Additionally, an ad hoc questionnaire written for this study was administered in order to obtain sociodemographic and academic information from the students, with the variables sex and educational level considered in this study.

### 2.3. Data Analysis

First, in order to analyze the relationships between the two main dimensions of the parenting model (acceptance/involvement and strictness/imposition) and the dimensions of self-regulated learning and academic stress evaluated, Pearson’s correlations were applied, controlling for the participants’ sex and educational levels.

Then, considering the scores in each dimension of the model, the families were classified according to the predominant parenting style that characterized them. To do so, taking into account the sex and age of the participants, the sample was dichotomized at the median (Pc50), considering both dimensions simultaneously, so that (1) families that scored above the median on both dimensions were defined as authoritative, (2) families that scored above the median on acceptance/involvement and below the median on strictness/imposition were classified as indulgent, (3) families that obtained scores below the median on acceptance/involvement and above the median on strictness/imposition were defined as authoritarian, and (4) families that scored below the median on both dimensions were classified as neglectful [8,9].

Once the families had been classified, a multivariate factorial design (2 × 3 × 4) was applied to each set of school adjustment criteria evaluated (self-regulated learning and academic stress), considering sex (male vs. female), educational level (CSE-1 vs. CSE-2 vs. PSE), and parenting style (authoritative vs. indulgent vs. authoritarian vs. neglectful) as independent variables to test possible interaction effects. Then, the main effects were analyzed, and the post-hoc Bonferroni test was applied (α = 0.05).

## 3. Results

### 3.1. Relationships of Acceptance/Involvement and Strictness/Imposition with Academic Stress and Self-Regulated Learning

Table 1 shows the results of the partial correlations (controlling for participants’ sex and educational level).

As the table shows, the acceptance/involvement dimension was significantly and negatively related to the academic stress dimensions of classroom interaction and family pressure. With regard to the self-regulated learning dimensions, significant and positive correlations were obtained with self-efficacy and metacognitive strategies, and significant and negative relationships were found with test anxiety, perception of lack of control over time, and procrastination.

The strictness/imposition dimension showed significant and positive relationships with the academic stress dimensions of classroom interaction and family pressure.

### 3.2. Previous Multivariate Factorial Designs

The MANOVA performed on the academic stress dimensions showed a significant effect of sex, Λ = 0.94, *F*(4, 409) = 6.70, *p* < 0.001, educational level, Λ = 0.85, *F*(8, 818) = 8.53, *p* < 0.001, and parenting style, Λ = 0.85, *F*(12, 1082.40) = 2.78, *p* < 0.01.

Likewise, significant effects of sex, Λ = 0.94, *F*(5, 406) = 5.10, *p* < 0.001, educational level, Λ = 0.94, *F*(10, 812) = 2.61, *p* < 0.01, and parenting style, Λ = 0.91, *F*(15, 1121.19) = 2.57, *p* < 0.01 were also obtained for the dimensions of self-regulated learning.

In both cases, no significant interaction effects were found; therefore, the main effects are analyzed below.

### 3.3. Parenting Styles, Academic Stress and Self-Regulated Learning

The ANOVA of the parenting styles and academic stress showed that there were significant differences in the dimensions of classroom interactions and family pressure. Bonferroni tests (α = 0.05) indicated that children from authoritative and authoritarian families obtained significantly higher means on both dimensions than children from indulgent families. Moreover, children from authoritative families obtained significantly higher means on family pressure than children from neglectful families (see Table 2).

In the ANOVA performed with self-regulated learning, significant differences were obtained in all the dimensions evaluated. The post-hoc Bonferroni tests (α = 0.05) showed that (1) children from authoritative and indulgent families obtained significantly higher scores on self-efficacy compared to children with authoritarian and neglectful parents, (2) children from authoritarian families obtained significantly higher means on exam anxiety than children from indulgent families, (3) children from authoritative families obtained significantly higher means on metacognitive strategies than children with authoritarian and neglectful parents, (4) children with authoritarian families obtained significantly higher means on perception of lack of control over the use of time compared to children with authoritative and indulgent parents. Likewise, children with neglectful parents scored higher than children from authoritative families, and (5) children from neglectful families obtained significantly higher scores on academic procrastination than children with authoritative and indulgent parents. Furthermore, children with authoritarian parents showed significantly higher scores than children from authoritative families (see Table 2).

### 3.4. Sex, Educational Level, Academic Stress and Self-Regulated Learning

Despite not being part of the main objective of this study, both demographic variables showed a significant effect on the school adjustment outcomes assessed.

On the one hand, the ANOVA carried out with sex and academic stress showed significant differences in the dimensions of academic overload and future expectations. In both cases, females obtained significantly higher scores than males. With regard to self-regulated learning, statistically significant differences were obtained in the dimensions of exam anxiety and self-regulation. Again, females obtained higher scores in both cases (see Table 3).

On the other hand, the ANOVA conducted with educational level and academic stress showed significant differences in all the dimensions evaluated. The Bonferroni tests (α = 0.05) indicated that (1) the CSE-2 and PSE students obtained significantly higher scores on the dimension of academic overload compared to the students in CSE-1, (2) the CSE-1 students showed significantly higher scores on classroom interactions compared to the CSE-2 and PSE students, (3) the CSE-2 students obtained significantly higher scores on family pressure than the PSE students, and (4) the CSE-2 students obtained significantly higher scores on the future expectations dimension than the CSE-1 students. With regard to the self-regulated learning dimensions, significant differences were obtained in the perception of lack of control and procrastination. The a posteriori tests indicated that (1) the CSE-2 students scored significantly higher on perception of lack of control over time than the CSE-1 and PSE students and (2) the CSE-2 students showed significantly higher scores on procrastination than the CSE-1 students (see Table 3).

## 4. Discussion

Taking into account the extensive literature addressing the relationship between parental socialization and children’s school adjustment using various classic indicators such as academic achievement, self-concept, and behavior problems in the classroom [57,61,68], as well as the importance of adopting a multidimensional perspective in the definition of school adjustment that includes other representative criteria [77], the objective of this study was to analyze which parenting style was related to better results on different cognitive, motivational, and behavioral dimensions of self-regulated learning and better results on academic stress in Spanish adolescents.

Based on the empirical evidence obtained in previous research on general psychosocial adjustment and, specifically, school adjustment, we expected that the parenting style mainly characterized by high warmth and low strictness, the indulgent style, would be related to the best results, or at least the same results as the parenting style characterized by high warmth and high strictness, the authoritative style, for the different criteria evaluated. In addition, it was also expected that this relationship would not vary according to the sex and educational level of the children.

The results obtained confirm this hypothesis. First, the performance of parents characterized by high use of practices related to showing affection, support, and emotional involvement, as well as communication and reasoning to modify poorly adjusted behaviors (high acceptance/involvement, an aspect that defines the indulgent and authoritative styles) was related to children’s greater perception of self-efficacy toward learning, greater use of metacognitive strategies, lower perception of lack of control over use of time, lower levels of academic procrastination, and less academic stress related to classroom interactions and family pressure. By contrast, the performance of parents characterized by high use of practices related to parental firmness and imposition to set limits on their children’s behavior (high strictness/imposition, an aspect that defines the authoritative and authoritarian styles) was related to higher levels of stress related to classroom interactions and family pressure.

Second, considering the four typologies derived from the confluence of the two orthogonal dimensions, the results indicated that (1) with regard to the different dimensions of self-regulated learning, the children of indulgent parents, like the children of authoritative parents, obtained better scores on self-efficacy, metacognitive strategies, perception of lack of control over time, and procrastination compared to children with authoritarian and neglectful parents. Likewise, children from indulgent families showed less test anxiety than children with authoritarian parents. (2) Regarding the academic stress dimensions, children from indulgent families showed less stress related to classroom interactions and family pressure, compared to children from authoritative and authoritarian families. Moreover, children with authoritarian parents showed greater academic stress related to family pressure than children with neglectful parents.

Therefore, these results suggest that the indulgent parenting style was related to better results, or at least equal to those obtained by children with authoritative parents, on the different school adjustment indicators evaluated. Although no significant differences were obtained between the indulgent and authoritative styles in the self-regulated learning dimensions evaluated, with both styles being ideal for fomenting important aspects of children’s learning process such as self-efficacy toward learning, use of academic self-regulation strategies, perception of control over the use of time, and low academic procrastination, there were significant differences in the academic stress dimensions, with children from indulgent families obtaining better scores than children with authoritative parents. This result shows the importance of high parental acceptance/involvement and the negative influence of high strictness/imposition on the stress that children can experience in their learning process [114]. Importantly, in line with some previous results in the Spanish context [14,17,57,58,60,62,115], but also extending evidence to the school context, in order to help children in their school success, parental strictness seems to be unnecessary (no differences were found between indulgent and authoritative in self-regulated learning outcomes) and may even be harmful (indulgent parenting was more protective than authoritative style against the academic stress related to classroom interactions and family pressure). Therefore, the best school student profile (adolescents with adequate learning strategies and lower levels of academic stress) was associated with indulgent parenting (warmth but not strictness).

Considering all of these aspects, the results of this study support previous empirical evidence obtained in Spain and other European and Latin America countries showing that the indulgent style was the form of parental performance related to the best results in children’s psychosocial adjustment [13,43,54,56,58,116]. They contribute further evidence about its suitability in relation to some relevant school adjustment criteria that are different from the ones traditionally employed (academic achievement, self-concept, and behavior problems in school) [57,66,74] and are much less addressed by the research in this area of study [89,90,101].

Moreover, the results of the study are also consistent with previous research that concluded that there are no significant variations in the best parenting style depending on demographic variables such as the sex and age of the children and the parents [10,11,12,13,17,50]. Thus, there were no significant interactions between the parenting styles and the participants’ sex or educational level, suggesting that the indulgent parenting style is related to the best results on the criteria evaluated, regardless of the students’ sex or educational level. With respect to sex and educational level, despite not being part of the main objective of the study, there were significant differences on the adjustment criteria evaluated depending on these demographic variables.

In this regard, the results obtained largely coincide with those pointed out by previous research that analyzed the effects of sex and educational level on the self-regulated learning and subjective wellbeing of adolescents in high school. Thus, as in previous research, females showed significantly higher scores than males in the use of academic self-regulation strategies [117,118,119,120,121,122,123], including time management strategies [113,124,125]. However, results from previous research on the effects of students’ sex and educational level on self-efficacy toward learning are controversial, with some studies showing homogeneous results between males and females in elementary school on self-efficacy toward mathematics and significant differences in favor of males at higher levels [126], whereas other studies highlighted the existence of differences between males and females on self-efficacy toward learning mathematics and significant differences in favor of males on verbal self-efficacy [117]. In this study, males and females showed homogenous levels of self-efficacy toward learning, coinciding with previous studies carried out in our context [112,127]. On the other hand, although previous research shows that students became more capable and efficacious in self-regulating their learning with age [128], it also highlighted the lack of significant differences between educational levels in high school in students’ scores on the dimensions of metacognitive strategies, lack of control over the use of time, and self-efficacy toward learning [112]. Some studies even pointed out that as this educational stage advances, students say that they use metacognitive strategies less [122,129] and that their academic and motivational involvement declines [108]. The results of this study moved in the direction highlighted in prior research, showing that all the educational levels demonstrated homogenous scores on dimensions related to metacognitive strategies and self-efficacy, whereas in CSE-2 students showed higher levels of lack of control over the use of time.

Additionally, the effects of students’ sex and educational level on test anxiety and academic procrastination also coincide with the results of previous research. Thus, the results of this study reveal that females showed significantly higher levels of test anxiety [81] and procrastinated less than males, although in the present study, the differences did not reach statistical significance [82,84,130,131]. Moreover, they also indicate that academic procrastination increased significantly throughout CSE and returned to its initial levels in PSE, indicating the development of effective strategies to overcome it and improvement with repeated practice [82,84].

In another vein, the conclusions of the large body of research analyzing the effects of sex and educational level on academic stress in adolescence still cannot be considered definitive [132]. Thus, the majority of studies that analyze this question highlighted that females showed higher levels of stress than males [98,133], although recent studies revealed that males and females presented similar levels of stress [93], or that females showed greater stress when facing certain stressors, e.g., related to academic achievement, whereas males did so when facing other stressors, e.g., conflicts with parents and teachers [94,96]. The results of this study point in the latter direction, given that females showed higher levels of stress than males on academic overload and future perspectives, whereas the same levels were found in females and males on family pressure and classroom interactions. However, studies that analyze the relationship between academic stress and educational levels show that higher levels of stress were expressed in transitional academic courses between educational stages, and that in these courses, females presented more stress than males [92,105,133,134]. Our results point out that stress levels in high school vary depending on the specific dimension analyzed. Thus, the stress generated by fulfilling the academic overload, family pressure, and future perspectives increased between CSE-1 and CSE-2, coinciding with the greater volume and difficulty of the academic tasks and greater family demands to finish CSE in the established periods in order to enter PSE. However, the opposite situation occurred in the dimension of classroom interactions, where increasingly satisfactory relationships and fewer conflicts with classmates and teachers were found as students advanced through this educational stage. Additionally, the levels of stress produced by academic overload and future perspectives were the same in PSE as in CSE-2, which is congruent with the argument presented above, whereas classroom interactions and family pressure declined. In any case, the results on the effect of sex and educational level on academic stress levels in high school must be considered in the design and development of school prevention and intervention programs in this area and in the evaluation of their efficacy [135].

As in all scientific research, this study has some positive aspects and some limitations. On the one hand, among the positive aspects, we can highlight that (1) the parenting styles were evaluated based on the classic model with two dimensions and four typologies, adequately representing all the theoretical distinctions the model offers and favoring the replication of empirical evidence supporting the optimal parenting style [136], (2) other criteria for children’s school adjustment were used, which were different from those traditionally used in the area of study (i.e., academic achievement, self-concept, and behavior problems in the classroom) [66,68,71], providing greater evidence about the suitability of the indulgent style for the adequate school adjustment of Spanish adolescents, and showing that this relationship was invariant across adolescents’ sex and educational level; (3) finally, the study was carried out taking into account the minimum sample size necessary to develop adequate statistical power (1 − β = 0.95) [102,103]. On the other hand, among the study limitations, we can highlight that (1) the classification of the families according to the performance pattern that characterized them in the parenting process was carried out using the children’s ratings. However, the results obtained are consistent with previous studies that used the parents’ ratings [55,72]. Moreover, there is empirical evidence suggesting that children’s ratings can be less biased by social desirability than those of the parents [137]. In addition, (2) the cross-sectional and non-experimental nature of the study did not allow us to rule out the effect of other variables on the relationships between parenting styles and children’s school adjustment, nor to conclude to what degree the better or worse adjustment of children is due to the parents’ parenting style, or whether the parenting style is partly determined by the children’s adjustment [138]. Further studies with longitudinal and quasi-experimental designs would contribute to overcoming these limitations [139].

In spite of this, the results obtained in the present study provide greater empirical evidence about the suitability of the performance pattern mainly based on high affect and low parental strictness, the indulgent style, for the adequate school adjustment of Spanish adolescents. Thus, these results have important practical implications concerning the family and its role in fostering the adequate functioning of their children in school. It is well known that family and school are the main contexts in which children and adolescents grow up [25,57,66,140,141]. Therefore, the family as the first socialization setting, and the school as the first formal institution in children’s education, have common responsibilities for children’s upbringing. In this sense, when active agents from both contexts work together, the functioning of children in school is improved [140]. According to this, it is important that families involve and participate actively in the teaching-learning process of their children, along with the school context [141] and, at the same time, that schools promote a series of measures in order to facilitate parental involvement and participation, such as the design of effective forms of bidirectional communication between school and family, including parents in school decisions or advising families on parenting issues to promote an adequate home environment to support children learning [140], paying special attention to fomenting the high use of all the parental practices related to demonstrations of affection, emotional support and involvement, communication, and dialogue with their children, in order to foster their adequate functioning in school.

## 5. Conclusions

In summary, the results obtained in the present study support previous evidence about the influence of different forms of parental performance on children’s psychosocial adjustment [8,15,18,62]. Although the results of the optimal parenting style show variations related to the cultural context [14,16], the empirical evidence obtained in the present study is consistent with results from previous research carried out in different countries in Latin America and Europe, including Spain, which conclude that the parenting style mainly characterized by high warmth and low strictness, the indulgent style, was the socialization strategy related to children’s best psychosocial adjustment results [3,44,46,47,67,71]. Specifically, the results of this research provide further evidence about the suitability of the indulgent style for children’s adequate school adjustment [57,66,74], using relevant criteria that are representative of the multidimensionality that characterizes school adjustment [77], such as the cognitive, motivational, and behavioral dimensions of self-regulated learning and academic stress, which are areas of special relevance in research and psychoeducational intervention due to their important influence on adolescents’ academic success [92,105,106]. Thus, this study shows the influence of parental socialization styles on children’s school adjustment, with the parenting style characterized by high warmth and low strictness being related to the best results on the different criteria considered, with this result being consistent across sex and educational level of children. Hence, the positive influence of parental involvement in children’s teaching-learning processes was highlighted [25], emphasizing the importance of high use of all the parenting practices related to demonstrations of warmth, emotional support and involvement, communication, and reasoning, as relevant factors in children’s adequate adjustment in the school context.

## Figures and Tables

**Table 1:** Partial correlation matrix for all the study variables, controlling for sex and educational level.

Variables	1	2	3	4	5	6	7	8	9	10
1. Acceptance/involvement	−									
2. Strictness/imposition	−0.09	−								
3. Academic overload	−0.04	0.08	−							
4. Classroom Interactions	−0.10 *	0.13 **	0.38 ***	−						
5. Family Pressure	−0.22 ***	0.24 ***	0.45 ***	0.38 ***	−					
6. Future Perspectives	0.01	0.01	0.50 ***	0.36 ***	0.35 ***	−				
7. Self-Efficacy	0.29 ***	−0.07	−0.05	−0.12 *	−0.23 ***	−0.03	−			
8. Test Anxiety	−0.11 *	0.09	0.31 ***	0.21 ***	0.32 ***	0.24 ***	−0.26 ***	−		
9. Metacognitive Strategies	0.24 ***	−0.01	0.13 **	0.03	−0.08	0.07	0.35 ***	−0.03	−	
10. Perception of control over time	−0.22 ***	0.08	0.28 ***	0.17 ***	0.38 ***	0.17 ***	−0.35 ***	0.27 ***	−0.23 ***	−
11. Procrastination	−0.21 ***	0.05	0.07	0.08	0.23 ***	0.06	−0.31 ***	0.20 ***	−0.42 ***	0.59 ***

* *p* < 0.05; ** *p* < 0.01; *** *p* < 0.001.

**Table 2:** Means, standard deviations (in parentheses), *F* values, and Bonferroni ^#^ test between parenting styles and stress and self-regulated learning dimensions.

School Adjustment	Parenting Styles				
	Authoritative	Indulgent	Authoritarian	Neglectful	
*Academic Stress*					*F*(3, 433)
Academic Overload	25.99 (5.77)	25.57 (4.91)	26.08 (5.67)	25.83 (5.38)	0.18
Classroom Interactions	15.30 ^1^ (4.76)	13.35 ^2^ (4.24)	15.51 ^1^ (4.82)	14.59 (4.10)	4.99 **
Family Pressure	11.68 ^1^ (4.06)	9.89 ^2^ (3.43)	13.10 ^1a^ (4.10)	11.14 ^b^ (4.30)	11.48 ***
Future Perspectives	12.74 (3.72)	12.26 (3.58)	12.03 (3.29)	12.65 (3.79)	0.93
*Self-regulated Learning*					*F*(3, 433)
Self-Efficacy	32.40 ^1^ (7.61)	33.78 ^1^ (6.97)	29.36 ^2^ (8.14)	29.06 ^2^ (7.45)	10.08 ***
Test Anxiety	11.29 (4.90)	10.20 ^2^ (4.91)	12.41 ^1^ (5.22)	11.14 (4.76)	3.44 *
Metacognitive Strategies	28.74 ^1^ (6.92)	28.07 (6.57)	25.94 ^2^ (7.61)	26.20 ^2^ (5.99)	4.58 **
Perception of Control Over Time	17.13 ^2b^ (5.26)	17.45 ^2^ (5.02)	19.58 ^1^ (5.40)	19.09 ^a^ (4.97)	5.92 **
Procrastination	14.63 ^2b^ (4.45)	14.80 ^2^ (4.40)	16.37 ^a^ (4.60)	16.61 ^1^ (3.93)	6.21 ***

* *p* < 0.05, ** *p* < 0.01, *** *p* < 0.001; ^#^ Differences are statistically significant (α = 0.05) when superscript differ from each other, 1 > 2; a > b.

**Table 3:** Means, standard deviations (in parentheses), and *F* value between sex and educational level and the stress and self-regulated learning dimensions.

School Adjustment	Sex			Educational level ^#^			
	Male	Female		CSE-1	CSE-2	PSE	
*Academic Stress*			*F*(1, 434)				*F*(2, 434)
Academic Overload	25.19 (5.22)	26.40 (5.56)	5.33 *	24.26 ^2^ (5.94)	26.69 ^1^ (5.00)	26.92 ^1^ (4.79)	11.58 ***
Classroom Interactions	14.48 (4.67)	14.87 (4.47)	0.77	15.89 ^1^ (4.69)	14.47 ^2^ (4.51)	13.44 ^2^ (4.05)	10.70 ***
Family Pressure	11.69 (3.88)	11.22 (4.32)	1.41	11.51 (3.93)	12.21 ^1^ (4.39)	10.35 ^2^ (3.83)	7.17 **
Future Perspectives	11.76 (3.43)	12.98 (3.67)	12.60 ***	11.91 ^2^ (3.62)	12.92 ^1^ (3.61)	12.50 (3.52)	3.15 *
*Self-regulated Learning*			*F*(1, 434)				*F*(2, 434)
Self-Efficacy	31.78 (7.50)	30.70 (8.00)	2.07	30.82 (8.78)	31.83 (7.08)	30.82 (7.24)	0.84
Test Anxiety	10.72 (5.09)	11.69 (4.87)	4.12 *	11.92 (5.19)	11.01 (5.04)	10.68 (4.56)	2.46
Metacognitive Strategies	26.07 (7.01)	28.27 (6.62)	11.37 **	26.99 (7.60)	26.72 (6.89)	28.39 (5.65)	2.27
Perception of control over time	18.42 (5.04)	18.15 (5.43)	0.29	17.43 ^2^ (5.27)	19.47 ^1^ (4.85)	17.82 ^2^ (5.48)	8.72 **
Procrastination	15.94 (3.83)	15.29 (4.85)	2.34	14.95 ^2^ (4.70)	16.40 ^1^ (4.33)	15.34 (4.04)	4.52 *

* *p* < 0.05, ** *p* < 0.01, *** *p* < 0.001; ^#^ Bonferroni test (α = 0.05). Differences are statistically significant when superscript differ from each other, 1 > 2.
